# Efficacy and safety of cangrelor as compared to ticagrelor in patients with ST-elevated myocardial infarction (STEMI): a systematic review and meta-analysis

**DOI:** 10.1186/s43044-024-00480-8

**Published:** 2024-04-16

**Authors:** Subhro Chakraborty, Debalina Sarkar, Shambo Samrat Samajdar, Pallab Biswas, Debasish Mohapatra, Saptarshi Halder, Mohammad Yunus

**Affiliations:** 1 Department of Cardiology, RGKar Medical College, HA 35, Sector 3, Saltlake CityKolkata, 700097 India; 2 Department of Endocrinology, RGKar Medical College, Kolkata, India; 3 School of Tropical Medicine Kolkata, Diabetes and Allergy-Asthma Therapeutics Speciality Clinic, Kolkata, India

**Keywords:** Cangrelor, Ticagrelor, High platelet reactivity (HPR), ST-elevated myocardial infarction (STEMI)

## Abstract

**Background:**

This systematic review and meta-analysis aimed to compare the efficacy and safety of cangrelor as compared to ticagrelor in patients with ST-elevated myocardial infarction (STEMI) who underwent percutaneous intervention.

**Methods:**

PubMed, Embase, Scopus, Web of Science, Cochrane CENTRAL, and ClinicalTrials.gov databases were searched for relevant head-on-comparison or swapping studies. The primary outcome was the rate of high platelet reactivity (HPR) at specific time intervals after stopping cangrelor infusion during the first 24 h. Secondary outcomes were the risks of thrombosis, all-cause mortality and bleeding. Pooled odds ratios (ORs) were calculated using random-effects models.

**Results:**

A total of 1018 studies were screened and eight were included in the analysis. There were four head-on-comparison studies and four swapping studies. There was no significant difference in the proportion of patients achieving a high platelet reactivity in swapping studies [OR, 0.71 (95% CI 0.04, 13.87), *p* = 0.82, *i*^2^ = 88%]. In head-on-comparison studies, PRU from Fig. 2B shows there was no significant reduction in high platelet reactivity [mean difference – 77.83 (95% CI − 238.84, 83.18), *p* < 0.001, *i*^2^ = 100%]. PRU results from (Fig. 2C) show a mean difference of 7.38 (95% CI − 29.74, 44.51), *p* < 0.001, *i*^2^ = 97%. There was no significant difference in the risks of thrombosis [OR, 0.91 (95% CI 0.20, 4.13), *p* = 0.81, *i*^2^ = 0%], all-cause mortality [OR, 3.52 (95% CI 0.44, 27.91), *p* = 0.24, *i*^2^ = 26%] and bleeding [OR, 0.89 (95% CI 0.37, 2.17), *p* = 0.93, *i*^2^ = 0%] between the two groups as revealed in the head-on-comparison studies.

**Conclusion:**

The efficacy and safety profiles of cangrelor and ticagrelor were similar in patients with STEMI.

**Supplementary Information:**

The online version contains supplementary material available at 10.1186/s43044-024-00480-8.

## Background

Coronary artery disease, an important etiology of premature mortality, is often manifested by ST-elevated myocardial infarction (STEMI). Oral antiplatelet therapy with activity against purinergic receptor (P2Y12) is beneficial in these patients in terms of mortality [1]. However, some of the main limitations of these drugs include the risk of bleeding, delayed onset of action, significant interindividual variability in the response, and extended duration of action that cannot be reversed if the need for hemostasis or emergency surgery arises [2]. Some of these drawbacks are counteracted by using intravenous (i.v.) agents.

Cangrelor, given through the i.v. route, is a directly acting receptor blocker [3]. It has a fast onset of action, and it has a better safety profile in terms of the reduction in the incidence of adverse events, such as myocardial infarction or stent thrombosis as compared to clopidogrel [4]. Cangrelor serves as a complementary therapy during PCI for patients who have not received adequate pretreatment with platelet P2Y12 receptor inhibitors before the procedure. Its usage necessitates a switch to an oral platelet P2Y12 receptor inhibitor. Transition plans are formulated based on the pharmacological properties of these inhibitors, pharmacodynamic studies and clinical trial findings. Cangrelor prevents the active metabolites of clopidogrel and prasugrel, both thienopyridines, from binding to the platelet P2Y12 receptor.

Ticagrelor, another reversible direct inhibitor of the platelet P2Y12 receptor, can be administered before or during cangrelor infusion without any interaction concerns. This is because cangrelor binds reversibly to the platelet P2Y12 receptor. Limitation of recovery of platelet function during the transition from parenteral to oral therapy is the rationale behind this strategy since the risk of acute stent thrombosis is minimized by limiting the recovery of the platelet’s function [5]. Another reason is because of the different half-lives, different sites and types of binding to the P2Y12 receptor between two drugs cangrelor and ticagrelor. Ticagrelor with a half-life of 6–12 h exceeds the time of duration of cangrelor infusion, meaning that the drug is available systemically to bind with the P2Y12 receptor even after stopping cangrelor and avoiding drug–drug interactions [6].

Several randomized controlled trials have evaluated the efficacy and safety of cangrelor as compared to ticagrelor (head-on or swapping studies) in patients with STEMI who underwent percutaneous intervention. However, there is no pooled evidence and, hence, this study was undertaken to quantitatively summarize the pooled evidence in this regard.

## Methods

### Search strategy

PubMed, Embase, Scopus, Web of Science, Cochrane CENTRAL, and ClinicalTrials.gov databases were searched from inception till October 31, 2023. Cross-references to previous meta-analyses on similar topics were also searched. Randomized controlled trials that compared the efficacy of cangrelor with ticagrelor in adult patients with STEMI undergoing percutaneous intervention were included. Studies that used other antiplatelet agents for percutaneous intervention were excluded. Observational studies, review articles, conference proceedings and case reports were also excluded. The suitability of the studies was determined by two independent authors. Any disagreement was resolved by discussing it with a third author. The authors retrieved the study abstracts and the full text of the selected articles. The Rayyan software was used for this purpose. Corresponding authors of relevant articles were contacted via email for any missing information. The search strategy is provided in Additional file 1: Table S1. Articles with full-text access were taken into consideration. Two types of studies (head-on comparison or swapping studies) of cangrelor versus ticagrelor were included. The primary outcome was the rate of high platelet reactivity (HPR) at specific time intervals after stopping cangrelor infusion during the first 24 h. Secondary outcomes were the risks of thrombosis, all-cause mortality and bleeding.

### Synthesis of data

Three authors independently carried out reviewing of the abstract and data extraction using a pre-formatted data extraction sheet. During data extraction, there were no assumptions or simplifications made.

### Data analysis

The Cochrane risk of bias tool-2 was used to assess the risk of bias. Descriptive statistics were used for insufficient data. Pooled mean difference and its 95% confidence interval (CI) were estimated for the continuous variables, and odds ratio (OR) and its 95% CI were estimated for the categorical variables. Heterogeneity was assessed by the *i*^2^ test with a 5% alpha error [7]. Low, medium and high levels of heterogeneity indicate 25%, 50% and 75% of heterogeneity. Meta-analysis was used for the primary and secondary outcomes by RevMan version 5.3 software. A *p*-value of < 0.05 was considered to be statistically significant for all tests.

### Ethics

This study was initiated after obtaining an exemption from review from the appropriate Institutional Ethics Committee.

## Results

**Fig. 1:**
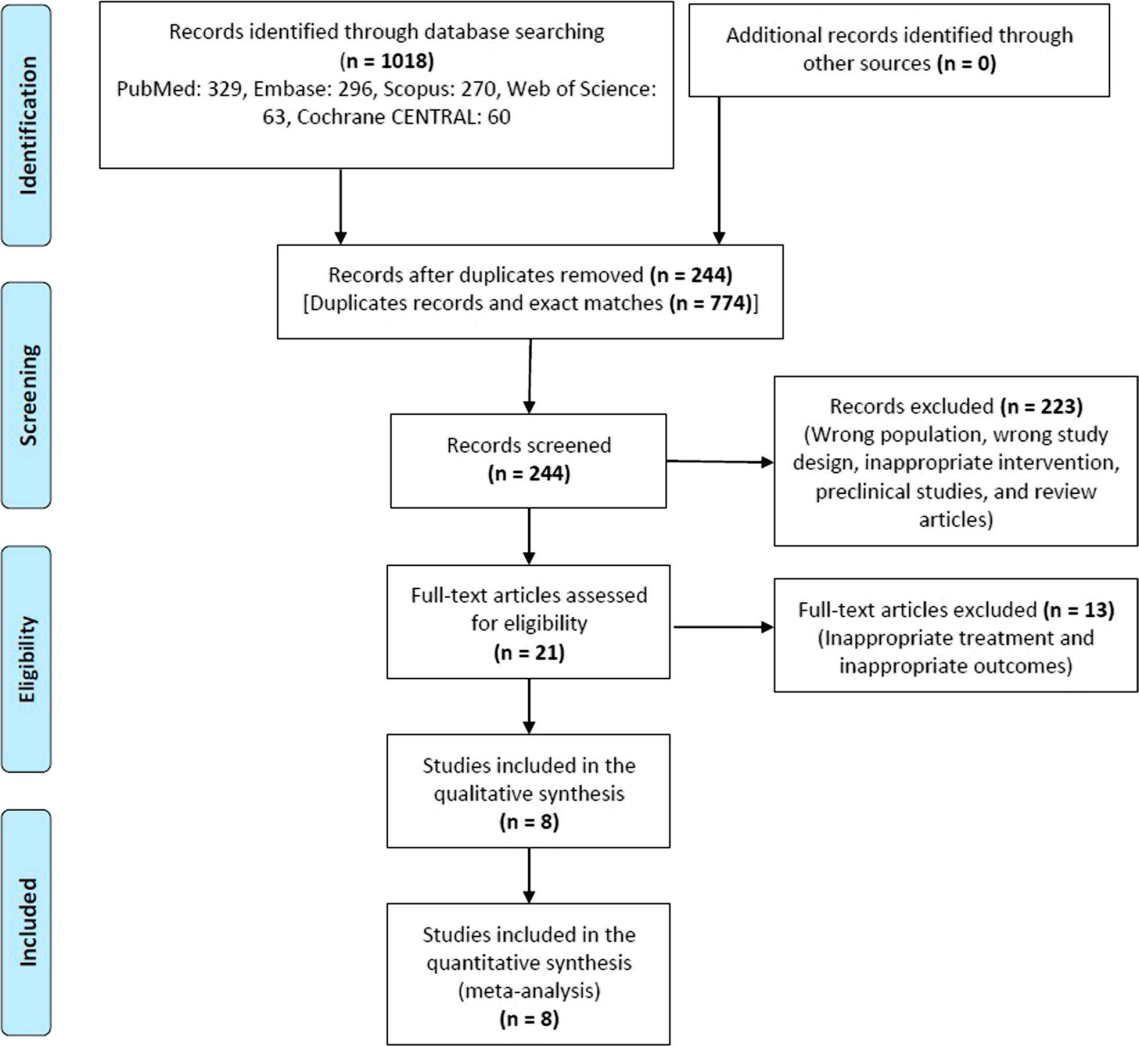
Study flow chart

A total of 1018 studies were screened and eight were included in the systematic review (Fig. 1) [8–15]. The individual characteristics of each study are enumerated in Table 1. There were 4 head-on-comparison studies and 4 swapping studies. Most of the studies have a low to medium risk of bias (Additional file 1: Figure s1).

**Table 1:** Characteristics of the included studies

Author, year	Study population	Age (years)	N	Male: Female	Primary Outcome	Cangrelor dosing regimen	Ticagrelor dosing regimen Comparator drug
Franchi F, 2023^*^	Patients with coronary artery disease	> 18	20	(9:11)	Measurement of PRU at different time points	30 mcg/kg bolus followed by 4 mcg/kg/min	Ticagrelor (180 mg loading dose)
Buchtele, 2020^*^	Adult survivors of out-of-hospital cardiac arrest with ST-segment elevation myocardial infarction (STEMI)	> 18	16	(13:3)	Rate of high platelet reactivity at different time points	30 mcg/kg bolus followed by 4 mcg/kg/min	Ticagrelor (180 mg oral loading dose of crushed ticagrelor)
Rossini, 2020^*^	Patients with previous PCI who were still on DAPT and undergoing nondeferrable surgery requiring DAPT discontinuation	> 18	24	(19:5)	Measurement of PRU at different time points	0.75 μg/kg/min infusion without a bolus	Ticagrelor ( 90 mg BD)
Mohammed, 2017^*^	STEMI patients undergoing primary PCI	> 18	32	(22:10)	Measurement of PRU at different time points	30 mcg/kg bolus followed by 4 mcg/kg/min	Ticagrelor (loading dose of 180 mg oral)
Franchi F, 2019^#^	STEMI Patients Undergoing Primary Percutaneous Coronary Intervention	> 18	50	(37:13)	Measurement of PRU at different time points	30 mcg/kg bolus followed by 4 mcg/kg/min	Ticagrelor (180-mg Loading dose of ticagrelor)
Ubaid S, 2019^#^	STEMI undergoing primary PPCI	> 18	100	(72:28)	Measurement of PRU at different time points	30 mcg/kg bolus followed by 4 mcg/kg/min	Ticagrelor (loading dose of 180 mg)
Scalia L, 2022^#^	STEMI undergoing primary PPCI	> 18	396		Composite of all-cause death, myocardial infarction (MI), definite or probable stent thrombosis and unplanned repeat revascularization within hospital discharge	30 mcg/kg bolus followed by 4 mcg/kg/min	Ticagrelor (loading dose of 180 mg)
Badreldin, 2017^#^	Patients undergoing PPCI	> 18	124	(84:40)	In hospital all-cause mortality and stent thrombosis	30 mcg/kg bolus followed by 4 mcg/kg/min	Ticagrelor (loading oral dose of 180 mg)

STEMI, ST-elevation myocardial infarction; PPCI, primary percutaneous coronary intervention

^#^Head-on Comparison studies

^*^Swapping studies

**Fig. 2:**
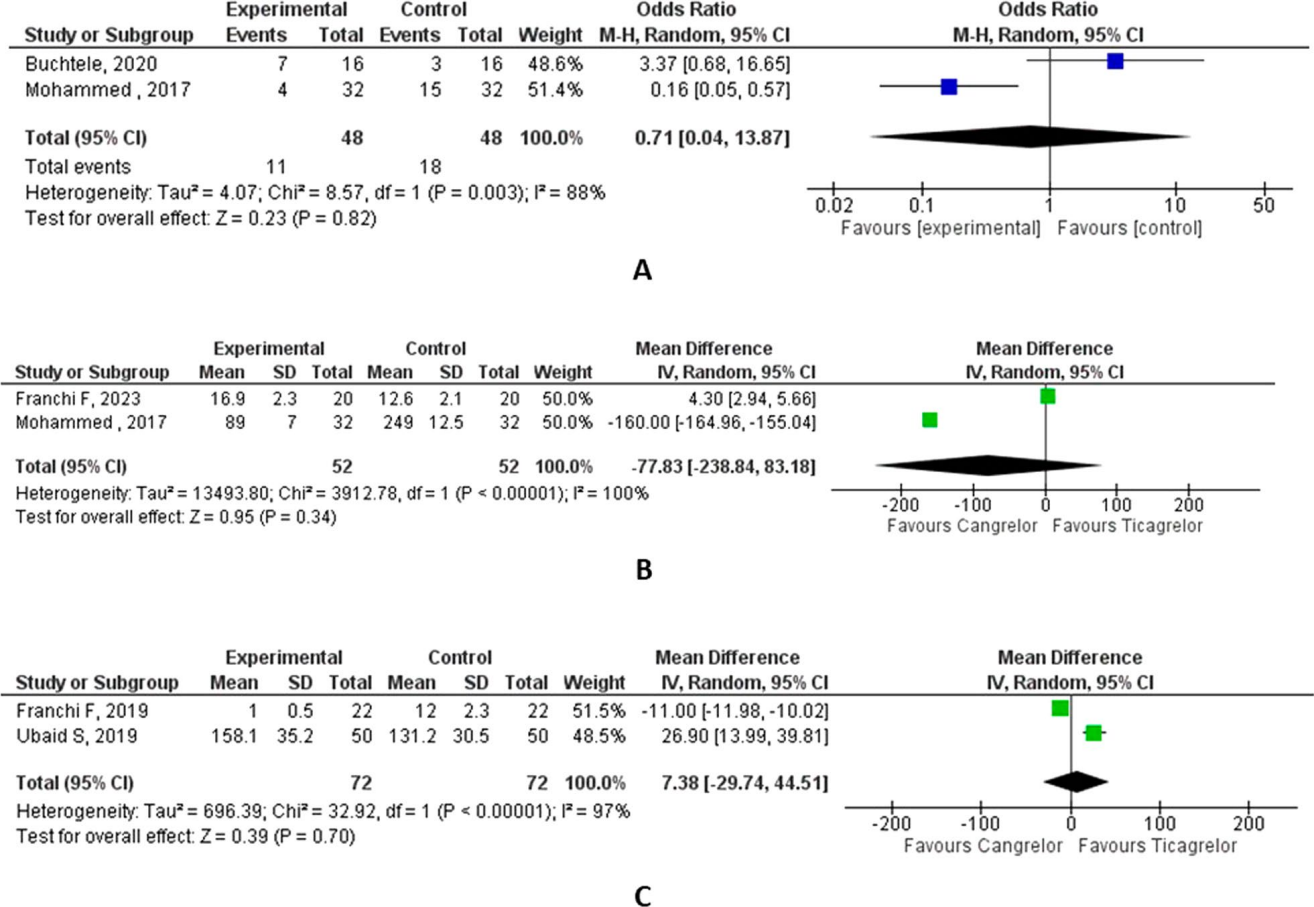
Effect of cangrelor vs. ticagrelor on proportion of patients achieving a high platelet reactivity (**A**) and platelet reactivity unit (**B**: swapping studies and **C**: head-on-comparison studies)

For the swapping studies, 92 patients were included having a male predominance. Patients with co-morbidities like diabetes mellitus, hypertension and hyperlipidemia are also included. There was no significant difference in the proportion of patients achieving a high platelet reactivity [OR, 0.71 (95% CI 0.04, 13.87), *p* = 0.82, *i*^2^ = 88%] (Fig. 2A). There was no significant reduction in high platelet reactivity [mean difference – 77.83 (95% CI − 238.84, 83.18), *p* < 0.001, *i*^2^ = 100%] (Fig. 2B). The study findings by Mohammad et al. showed that the level of P2Y12 inhibition is consistent between the ticagrelor-given groups in prehospital and cath laboratories. In the included sample size of 32 patients, cangrelor in combination with ticagrelor results in consistent P2Y12 inhibition and may bridge the gap till oral P2Y12 inhibitors achieve the effect [8].

**Fig. 3:**
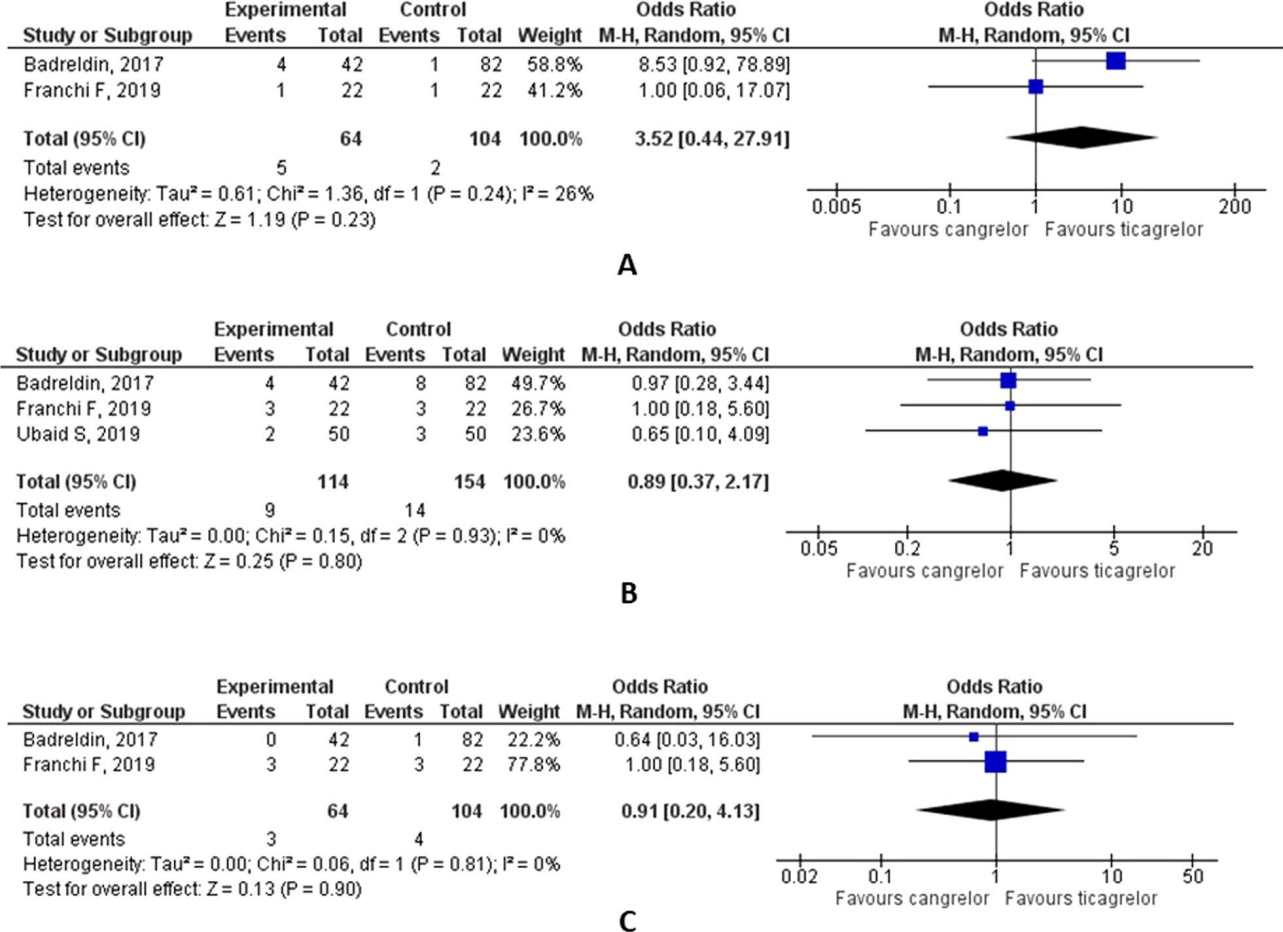
Effect of cangrelor vs. ticagrelor on the risks of all-cause mortality (**A**), bleeding (**B**) and thrombosis (**C**)

Franchi et al. (2023) showed that platelet reactivity units were similar between cangrelor and placebo (16.9 vs. 12.6; LSM difference: 4.3; 95% CI − 28.6 to 37.3; *P* for superiority = 0.797), with the upper margin of the 95% CI of LSM difference below the 45 PRU noninferiority margin and meeting the primary noninferiority endpoint [9]. For the four head-on studies, 670 patients were included having a male predominance in all the included four studies. Patients with comorbidities like diabetes mellitus, hypertension and hyperlipidemia are also included. PRU results from (Fig. 2C) show a mean difference of 7.38 (95% CI − 29.74, 44.51), *p* < 0.001, *i*^2^ = 97%. There was no significant difference in the risks of thrombosis [RR, 0.91 (95% CI 0.20, 4.13), *p* = 0.81, *i*^2^ = 0%], all-cause mortality [RR, 3.52 (95% CI 0.44, 27.91), *p* = 0.24, *i*^2^ = 26%] and bleeding [RR, 0.89 (95% CI 0.37, 2.17), *p* = 0.93, *i*^2^ = 0%] between the two groups (Fig. 3). In the study by Ubaid et al., the platelet reactivity unit was measured during the inflation of the coronary balloon, and the cangrelor produced greater P2Y12 inhibition [10]. This difference was no longer apparent at 4 h and 24–36 h after the study drug administration.

In the study by Franchi et al., cangrelor treatment was associated with reduced P2Y12 reaction units as early as 5 min after bolus, which persisted during the drug infusion, including at 30 min (63 [32–93] versus 214 [183–245] in the placebo group.

## Discussion

The purpose of this study was to evaluate the efficacy and safety of cangrelor as compared to ticagrelor in patients with STEMI. There was no statistically significant difference in terms of the efficacy (high platelet reactivity and PRU) and safety (thrombosis, all-cause mortality and bleeding) outcomes between cangrelor and ticagrelor arm in both head-on-comparison and swapping studies.

Our findings are similar to those of Westman et al. [16] which reported no differences between cangrelor arm and clopidogrel arm about cardiovascular death (OR 1.01 [CrI 0.23–4.39]), myocardial infarction (OR 0.94 [CrI 0.69–1.25]), major adverse cardiac events (OR 0.91 [CrI 0.69–1.18]), stent thrombosis (OR 0.66 [CrI 0.37–1.19]) or major bleeding (OR 1.52 [CrI 0.79–2.98]). In terms of the difference in PRU, there was no significant difference between cangrelor and ticagrelor arms. This finding is in contrast with that of the CANTIC study done by Franchi et al. [14], which noticed treatment with cangrelor maintained lower levels of platelet reactivity compared with placebo (63 [32–93] versus 214 [183–245]; mean difference, 152; 95% CI 108–195; *P* < 0.001) at 30 min and until the end of its 2-h infusion.

The interaction between ticagrelor and cangrelor, both of which are reversible direct inhibitors of the platelet P2Y12 receptor, is notably absent. This lack of interaction allows for the administration of ticagrelor either before or during the infusion of cangrelor without concerns regarding interference or adverse effects. This characteristic is advantageous in clinical settings where immediate antiplatelet effects are required, such as during percutaneous coronary intervention procedures. This ensures the use of ticagrelor alongside cangrelor, ensuring effective platelet inhibition.

The hypothesis of potent and quick onset P2Y12 inhibition during percutaneous intervention greatly protects from periprocedural ischemic events [17]. A timely PCI is essential but some patients develop limitations of microvascular perfusion though there is restoration of blood flow [18]. This shows that the role of platelets is important since they contribute to adverse events like thrombosis and embolization and the presence of large infarcts. This is the reason for measuring platelet reactivity unit, and there was no statistical significance between ticagrelor and cangrelor groups. In a previous study, it was found that compliance with any P2Y12 inhibitor therapy was related to a 21% lower relative risk of major adverse cardiovascular events as compared to noncompliance with the P2Y12 inhibitor therapy [19]. The adherence pattern is important concerning analyzing clinical outcomes. The gap needs to be bridged in patients with STEMI undergoing primary PCI since the study results did not show efficacy although ticagrelor is given in crushed form within a short time. [20]

However, ticagrelor was found to be a safe anti-platelet agent with less incidence causing bleeding as compared to prasugrel in line with the findings of the network meta-analysis by Fei et al [21]. In this study [21], prasugrel was found to be more beneficial in reducing major adverse cardiovascular events, MI and definite or probable stent thrombosis but lead to higher risk of major and minor bleeding. Ticagrelor and cangrelor reduced definite or probable stent thrombosis and cardiovascular mortality with cangrelor causing more thrombolysis in myocardial infarction minor bleeds in comparison with clopidogrel.

Dual antiplatelet therapy with aspirin and clopidogrel after acute coronary syndrome is related to a reduction in bleeding complications. Monitoring of bleeding events is necessary. In the TRITON-TIMI (therapeutic outcomes by optimizing platelet inhibition with prasugrel thrombolysis in myocardial infarction) trial, there was a reduction in ischemic endpoints (viz. death from the vascular cause, MI, stroke) in patients treated with ticagrelor [22, 23]. There was a higher incidence of bleeding complications in comparison with clopidogrel. The presence of bleeding is a major issue since it is one of the frequent noncardiac complications with an adverse impact on prognosis [24–27]. Dual antiplatelet therapy for one year is considered as standard of care for patients with acute coronary syndrome. Compliance is also one major concern and around 30% of patients having ACS discontinue one medication within one month [28]. A fixed drug combination of aspirin and clopidogrel was found to improve adherence and help in preventing adverse events like dyspnea and bleeding [29, 30].

The studies included in the analysis exhibit several limitations. The low number of studies and small sample sizes may impact the robustness of the findings, and the results could potentially change with the availability of more extensive studies. During the review process, some outcomes of interest had limited data availability, leading to their exclusion or inability to be included in the meta-analysis. Substantial heterogeneity in most outcomes is another limitation, and this heterogeneity can be attributed to variations in the study population, the duration and severity of NSTEMI, the use of different concurrent medications across the studies and variations in the disease and duration of drug therapy.

## Conclusion

The efficacy and safety profiles of cangrelor and ticagrelor were similar in patients with STEMI. Oral ticagrelor can be used as bridge therapy in patients requiring the infusion of cangrelor and with the benefit of no drug–drug interactions between the two drugs, both can be given with monitoring of bleeding complications and the issues which can happen due to lack of medication adherence should be borne in mind by the clinicians for the better clinical outcome.

### Supplementary Information

**Additional file 1**. Search strategy and supplementary results.

## Data Availability

Data will be available on reasonable request.

## Abbreviations

CI: Confidence interval
HPR: High platelet reactivity
i.v.: Intravenous
OR: Odds ratio
P2Y12: Purinergic receptor
STEMI: ST-elevated myocardial infarction
TRITON-TIMI: Therapeutic outcomes by optimizing platelet inhibition with prasugrel thrombolysis in myocardial infarction
