# Supplementary material for: Efficacy and safety of cangrelor as compared to ticagrelor in patients with ST-elevated myocardial infarction (STEMI): a systematic review and meta-analysis
Source: Egypt Heart J. 2024 Apr 16;76:48. doi: 10.1186/s43044-024-00480-8 (PMC11021388; doi:10.1186/s43044-024-00480-8)
Supplement: Supplementary file 1 — Additional file 1. Search strategy and supplementary results. [file 43044_2024_480_MOESM1_ESM.docx]

**Supplementary Materials**

**Table s1.** Literature search strategy.

| Patients | PubMed, Embase, Web of Science, Scopus, and Cochrane CENTRAL | ST Segment Elevation Myocardial Infarction[Mesh] OR ST Elevated Myocardial Infarction[Mesh] OR STEMI[Mesh] |
| --- | --- | --- |
|  | Clinical[Trials.gov](http://trial.gov/) | Cangrelor OR ticagrelor AND ST Segment Elevation Myocardial Infarction |
| Intervention | PubMed, Embase, Web of Science, Scopus, and Cochrane CENTRAL | N(6)-(2-methylthioethyl)-2-(3,3,3-trifluoropropylthio)-5'-adenylic acid monoanhydride with dichloromethylenebis(phosphonic acid)[Mesh] OR cangrelor tetrasodium[Mesh] OR AR C69931MX[Mesh] OR AR-C69931MX[Mesh] OR Kengreal[Mesh] OR ticagrelor[Mesh] OR Brilique[Mesh] OR AZD 6140 [Mesh] OR AZD6140[Mesh] OR AZD-6140[Mesh] OR Brilinta[Mesh] OR 3-(7-((2-(3,4-Difluorophenyl)cyclopropyl)amino)-5-(propylthio)-3H-(1-3)-triazolo(4,5-d)pyrimidin-3-yl)-5-(2-hydroxyethoxy)cyclopentane-1,2-diol[Mesh] |
|  | Clinical[Trials.gov](http://trial.gov/) | Cangrelor OR ticagrelor AND ST Segment Elevation Myocardial Infarction |

| **Author, year** | **Bias arising from the randomization process** | **Bias due to deviations from intended interventions** | **Bias due to missing outcome data** | **Bias due to measurement of the outcome** | **Bias in selection of the reported result** | **Overall bias** |
| --- | --- | --- | --- | --- | --- | --- |
| Franchi F, 2023 |  |  |  |  |  |  |
| Buchtele, 2020 |  |  |  |  |  |  |
| Rossini, 2020 |  |  |  |  |  |  |
| Mohammed, 2017 |  |  |  |  |  |  |
| Franchi F, 2019 |  |  |  |  |  |  |
| Ubaid S, 2019 |  |  |  |  |  |  |
| Scalia L, 2022 |  |  |  |  |  |  |
| Badreldin, 2017 |  |  |  |  |  |  |

| **Index** | |
| --- | --- |
|  | Low risk of bias |
|  | Moderate risk of bias |
|  | High risk of bias |

**Figure s1.** Risk of bias assessment following the revised Cochrane risk-of-bias 2 tool.
